# Current Research on Probiotics and Fermented Products

**DOI:** 10.3390/foods13091406

**Published:** 2024-05-03

**Authors:** Yushi Dong, Mohan Li, Xiqing Yue

**Affiliations:** College of Food Science, Shenyang Agricultural University, Shenyang 110866, China; 2022200035@stu.syau.edu.cn

The history of probiotics and fermented products has evolved over millennia [1,2]. Traditional food fermentation benefited from people’s appreciation of its unique flavor and texture, while modern fermentation technology places more emphasis on the production of bioactive metabolites during fermentation [3,4,5]. These metabolites can enhance the bioavailability of functional components in food and offer numerous health advantages [6,7,8]. Modern technology relies on the selection of specific starter cultures to achieve the optimally expected target, such as improving the flavor, texture, bioavailability, or preventing specific diseases [9,10,11,12,13]. In recent years, an increasing number of studies have revealed the beneficial roles of probiotics and fermented products in human wellness [14,15,16]. Therefore, their applications in food, medicine, and other functional products has garnered growing attention. However, there are still many aspects of these probiotics and fermented products that have not been fully elucidated [17]. These include the identification and screening of strains in fermented products, optimizing of the fermentation process, understanding the succession law of flora in fermented products, exploring the correlation between microbiota and flavor in fermented products, understanding their roles in the preparation of various fermented foods, their varying tolerances to temperature, pH, and other environmental factors, improvement strategies, and the detailed molecular mechanisms and key active ingredients of these probiotics and fermented products in health regulation functions, among others [18,19,20].

In an era where the quest for optimal health intersects with the science of food and nutrition, the exploration of probiotics and fermented products emerges as a pivotal domain. This Special Issue of *Foods*, titled “Current Research on Probiotics and Fermented Products”, offers a comprehensive dive into the current state and future trajectory of this vibrant field. With three review papers and seven research articles, this collection illuminates the multifaceted roles of microorganisms in foods, health, and diseases, underscoring the ancient art of fermentation as a cornerstone of modern food and nutrition science.

Lactic acid bacteria (LABs) are beneficial microbes known for their health-promoting properties. LABs are well known for their ability to produce substantial amounts of bioactive compounds during fermentation. Peptides, exopolysaccharides (EPS), bacteriocins, some amylases, proteases, lipase enzymes, and lactic acid are the most important bioactive compounds generated by LAB activity during fermentation. Furthermore, the product produced by LAB is dependent on the type of fermentation used. LABs derived from the genera *Lactobacillus* and *Enterococcus* are the most popular probiotics at present. Consuming fermented foods has previously been associated with several health-promoting benefits, such as antibacterial activity and modulation of the immune system. Furthermore, functional food implementations lead to the application of LABs in therapeutic nutrition, such as prebiotic, immunomodulatory, antioxidant, antitumor, and blood-glucose-lowering actions. Understanding the characteristics of LABs from various sources, and its potential as a functional food, is crucial for therapeutic applications. The review by Hakim et al. presents an overview of functional food knowledge regarding interactions between LABs isolated from dairy products (dairy LABs) and fermented foods, as well as the prospect of functioning LABs in human health. Finally, the health advantages of bioactive compounds from LABs are emphasized.

Plant-based drinks have garnered significant attention as viable substitutes for traditional dairy milk, providing options for people who are lactose intolerant or allergic to dairy proteins and those who adhere to vegan or vegetarian diets. In recent years, the demand for plant-based drinks has expanded rapidly. Each variety has unique characteristics in terms of flavor, texture, and nutritional composition, offering consumers a wide range of choices tailored to meet individual preferences and nutritional needs. In the review by Xie et al., they aimed to provide a comprehensive overview of the various types of plant-based beverages and explore potential considerations, including their nutritional compositions, health benefits, and processing technologies, as well as the challenges facing the plant-based beverage processing industry. This review explores the scientific evidence supporting the consumption of plant-based beverages, discusses their potential role in meeting dietary requirements, and addresses current limitations and concerns about their use. In conclusion, this review illuminates the growing importance of plant-based drinks as sustainable and nutritious alternatives to dairy milk, and assists people in making informed choices about their dietary habits, expanding the potential applications for plant-based drinks, and providing the necessary theoretical and technical support for the development of a plant-based drink processing industry.

Functional dyspepsia (FD) is a common functional gastrointestinal disorder. Pathophysiology remains poorly understood; however, alterations in the small intestinal microbiome have been observed. Current treatments for FD with drugs are limited, and there are certain safety problems. A class of active probiotic bacteria can control gastrointestinal homeostasis, nutritional digestion and absorption, and energy balance, when taken in certain doses. Probiotics play many roles in maintaining the intestinal microecological balance, improving the intestinal barrier function, and regulating the immune response. The presence and composition of intestinal microorganisms play a vital role in the onset and progression of FD, and serve as a critical factor for both regulation and potential intervention regarding the treatment of this condition. Thus, there are potential advantages to alleviating FD by regulating the intestinal flora using probiotics that target intestinal microorganisms. The review by Shen et al. summarizes the progress of probiotic research in improving FD by regulating intestinal flora and provides a reference basis for probiotics to improve FD.

Fermented dairy foods, such as yogurt, exhibit some beneficial effects on consumers, including relieving symptoms of hypertension. The study by Yu et al. aims to obtain fermented dairy products from a co-starter that has a great flavor and the auxiliary function of reducing blood pressure after long time consumption. Commercial starter cultures composed of *Lactobacillus delbrueckii* subsp. *bulgaricus* CICC 6047 and *Streptococcus thermophilus* CICC 6038 were combined with *Lactobacillus plantarum* strains Y44, Y12 and Y16, respectively, as a combined starter culture to ferment a mix of skim milk and soybean milk. The fermented milk produced using the combined starter culture mixed with *L. plantarum* Y44 showed an inhibitory activity of the angiotensin converting enzyme (ACE) (53.56 ± 0.69%). Some peptides that regulate blood pressure were released in fermented milk, such as AMKPWIQPK, GPVRGPFPII, LNVPGEIVE, NIPPLTQTPV, and YQEPVL. In spontaneously hypertensive rat (SHR) oral administration experiments compared to the gavage unfermented milk group, gavage feeding of SHR with fermented milk produced using the combined starter culture mixed with *L. plantarum* Y44 significantly reduced SHR blood pressure after long-term intragastric administration, shown with the systolic blood pressure (SBP) and diastolic blood pressure (DBP) decreasing by 23.67 ± 2.49 mmHg and 15.22 ± 2.62 mmHg, respectively. Furthermore, the abundance of short-chain fatty acids (SCFA), bacterial diversity in the gut microbiota, and SCFA levels, including acetic acid, propionic acid, and butyric acid in the feces of SHRs, were increased by oral administration of fermented milk produced using the combined starter culture containing *L. plantarum* Y44. Furthermore, the ACE-angiotensin II (Ang II)-angiotensin type 1 (AT 1) axis was negatively regulated, the angiotensin-converting enzyme 2 (ACE 2)-angiotensin(1-7) (Ang1-7)-Mas receptor axis of SHRs was positively regulated, and then the RAS signal was rebalanced. Fermented milk obtained from the combined starter culture shows the potential to be a functional food with antihypertensive properties.

The creation of functional foods through the enrichment of new foods with probiotic organisms or spores is a promising approach. The study by Suwanangul et al. investigates the use of encapsulating agents to establish a synbiotic relationship with *Bacillus coagulans* (TISTR 1447). Various proportions of wall materials, such as skim milk powder, maltodextrin, and cellulose acetate phthalate (represented as SMC1, SMC3, SMC5 and SMC7), were examined. In all formulations, 5% inulin was included as a prebiotic. The research evaluated their impact on cell viability and bioactive properties during both the spray-drying process and in vitro gastrointestinal digestion. The results demonstrate that these encapsulating agents efficiently protect *B. coagulans* spores during the spray drying process, resulting in spore viability exceeding 6 log CFU/g. In particular, SMC5 and SMC7 displayed the highest spore viability values. Furthermore, SMC5 showed the most notable antioxidant activity, encompassing DPPH, hydroxy radical, and superoxide radical scavenging, as well as significant antidiabetic effects through the inhibition of α-amylase and α-glucosidase. Furthermore, during simulated gastrointestinal digestion, both SMC5 and SMC7 exhibited a slight reduction in spore viability during the 6 h simulation. Consequently, SMC5 was identified as the optimal condition for synbiotic production, providing protection to *B. coagulans* spores during microencapsulation and gastrointestinal digestion while maintaining bioactive properties after encapsulation. Synbiotic microcapsules containing SMC5 showed a remarkable positive impact, suggesting its potential as an advanced food delivery system and a functional ingredient for various food products.

Wild artisanal cultures, such as a symbiotic culture of bacteria and yeast (SCOBY) and water kefir grains (WKG), represent a complex microorganism consortium composed of yeasts and lactic and acetic acid bacteria, with large strains of diversity and abundance. Fermented products (FP) obtained by the contribution of the microbiome can be included in functional products, due to their metabiotics (pre, pro, post and paraprobiotics) resulting from complex and synergistic associations, as well as due to metabolic functionality. In the study performed by Pihurov et al., consortia of both SCOBY and WKG were involved in the cofermentation of a newly formulated substrate, which was further analyzed with the aim of increasing the postbiotic composition of the FPs. Plackett–Burman (PBD) and Response Surface Methodology (RSM) techniques were used for the experimental designs to select and optimize several parameters that influence the lyophilized starter cultures of SCOBY and WKG activity as a multiple inoculum. Tea concentration (1–3%), sugar concentration (5–10%), raisins concentration (3–6%), SCOBY lyophilized culture concentration (0.2–0.5%), WKG lyophilized culture concentration (0.2–0.5%), and fermentation time (5–7 days) were considered the independent variables for mathematical analysis and optimization of fermentation conditions. Antimicrobial activity against *Bacillus subtilis* MIUG B1, *Staphylococcus aureus* ATCC 25923, *Escherichia coli* ATCC 25922, and *Aspergillus niger* MIUG M5, antioxidant capacity (DPPH), pH and total acidity (TA) were evaluated as responses. The rich postbiotic bioactive composition of the FP obtained under optimized biotechnological conditions highlighted the usefulness of the artisanal cocultures through their symbiotic metabolic interactions for the improvement of bioactive potential.

To develop a new or better combination of probiotics and/or probiotic-prebiotics for a healthy functional food ingredient or a remedial agent to treat or prevent obesity, the potential antiobesity efficacy of *Lactobacillus rhamnosus* BST-L.601 and its fermented product (named SPY) with mashed sweet potato paste were investigated using 3T3-L1 preadipocytes and obese mice induced by the high fat diet (HD) by Kang et al. SPY (0–0.5 mg/mL) dose dependently and significantly reduced lipid accumulation and TG content and the expression of adipogenic markers (C/EBPα, PPAR-γ, and aP2) and fatty acid synthetic pathway proteins (ACC and FAS) in adipocytes 3T3-L1 adipocytes, demonstrating that SPY suppresses adipocyte differentiation and lipogenesis. Oral administration of SPY (4 × 107 CFU/kg body weight) to HD-induced obese mice for 12 weeks significantly reduced body and liver weight, adipocyte size, and epididymal, visceral, and subcutaneous fat tissues weight. SPY was more effective in decreasing body weight gain in HD mice than in treatment with BST-L.601 alone. The administration of SPY or BST-L.601 also reduced the serum level of total cholesterol and LDL cholesterol and leptin secretion to a similar level. These results revealed that both SPY and BST-L.601 effectively suppress HD-induced adipogenesis and lipogenesis, suggesting that these materials would be useful in the functional foods industry to improve and/or prevent obesity.

*Lacticaseibacillus paracasei* (formerly Lactobacillus paracasei) is a nomadic LAB that inhabits a wide variety of ecological niches, from fermented foodstuffs to host-associated microenvironments. Many isolated strains of *L. paracasei* have been used as single-strain probiotics or as part of a symbiotic consortium within formulations. The study proposed by Moiseenko et al. contributes to the exploration of different strains of *L. paracasei* derived from non-conventional isolation sources, the traditional South African fermented drink mahewu (strains MA2 and MA3) and kefir grains (strains KF1 and ABK). The microbiological, biochemical, and genomic comparative analyses performed on the studied strains demonstrated a correlation between the properties of the strains and their isolation source, suggesting the presence of at least partial adaptation of the strain to the isolation environments. Furthermore, for the studied strains, antagonistic activities against common pathogens and against each other were observed, and the ability to release bioactive peptides with antioxidant and angiotensin I-converting enzyme inhibitory properties (ACE-I) during milk fermentation was investigated. The results obtained may be useful for a deeper understanding of the nomadic lifestyle of *L. paracasei*, and for the development of new starter cultures and probiotic preparations based on this LAB in the future.

Greek yogurt is a strained yogurt with a high protein content that provides nutritional benefits. To enhance the functional benefits of Greek yogurt, Yang et al. formulated Greek yogurt with various combinations of probiotic LAB (*Streptococcus thermophilus*, *Lactobacillus bulgaricus*, *Lactobacillus gasseri* BNR17, and *Lactobacillus plantarum* HY7714) by Yang et al. Effects of probiotic LAB were compared on the quality, sensory and microbiological characteristics of Greek yogurt. Among the samples, Greek yogurt fermented with *S. thermophilus* and *L. bulgaricus* showed the highest changes in pH and titratable acidity during 21 days of storage at 4 °C. Greek yogurt fermented with *L. plantarum* HY7714 had a higher viscosity than other samples. Greek yogurt fermented with S. thermophilus, *L. bulgaricus*, *L. gasseri* BNR17 and *L. plantarum* HY7714 showed superior physicochemical properties, and received the highest preference score from sensory evaluation among samples. In general, the enterohaemorrhagic *Escherichia coli* (EHEC) population was more effectively reduced in fermented Greek yogurt with probiotic LAB than in commercial Greek yogurt during storage at 4, 10, and 25 °C. Therefore, the addition of *L. gasseri BNR17* and *L. plantarum* HY7714 as starter cultures could improve the microbial safety of Greek yogurt and the sensory acceptance of consumers.

Taking into account the need for functional foods and the use of by-products of the food industry, Mendonça et al. developed a potentially functional ice cream using soy extract, soy kefir, and dehydrated jaboticaba peel. Five ice creams were made using soy kefir (K) and soy extract (S): (1) GS-100% S; (2) GK1-75% S/25% K; (3) GK2-50% S/50% K; (4) GK3-25% S/75% K; and (5) GK-100% K. The products were evaluated by physicochemical, microbiological, and sensory analyses. The addition of kefir was found to increase the acidity of the products. The concentrations of total phenolic compounds in the kefir formulations were approximately ten times higher than those of the GS formulation. All products presented concentrations of thermotolerant coliforms < 3 NMP/g and absence of *Salmonella* ssp. The viability of *Lactobacillus* ssp., *Streptococcus* spp., and *Bifidobacterium* ssp. was greater than 10 log CFU/g during the storage period. GS and GK1 formulations had the lowest scores, while GK ice cream was preferred. The formulations showed distinct sensory profiles in the CATA and ice cream with 100% kefir was associated with desirable attributes. The ice creams exhibited microbiological and sensory characteristics that meet the expectations of the target audience of the product.

The investigation into probiotics and fermented products represents an interdisciplinary endeavor, integrating scientific inquiry, food science innovation, and societal impact. Ongoing research efforts are uncovering new advancements and potential in probiotics and fermented foods, offering insights that go beyond traditional fermentation practices. As we delve further into this realm of complexity, the opportunities for creativity, health promotion, and sustainability in probiotics and fermented products continue to expand and remain ready for exploration.
